# A Cardiac Early Warning System with Multi Channel SCG and ECG Monitoring for Mobile Health

**DOI:** 10.3390/s17040711

**Published:** 2017-03-29

**Authors:** Prasan Kumar Sahoo, Hiren Kumar Thakkar, Ming-Yih Lee

**Affiliations:** 1 Department of Computer Science and Information Engineering, Chang Gung University, Taoyuan City 33302, Taiwan; pksahoo@mail.cgu.edu.tw (P.K.S.); D0221015@stmail.cgu.edu.tw (H.K.T.); 2 Division of Cardiology, Department of Internal Medicine, Chang Gung Memorial Hospital, Taoyuan City 33305, Taiwan; 3 Graduate Institute of Medical Mechatronics, Center for Biomedical Engineering, Chang Gung University, Taoyuan City 33302, Taiwan

**Keywords:** Mobile Health (mHealth), Body Area Network (BAN), Coronary Heart Disease (CHD), Electrocardiography (ECG), Seismocardiography (SCG), warning system

## Abstract

Use of information and communication technology such as smart phone, smart watch, smart glass and portable health monitoring devices for healthcare services has made Mobile Health (mHealth) an emerging research area. Coronary Heart Disease (CHD) is considered as a leading cause of death world wide and an increasing number of people die prematurely due to CHD. Under such circumstances, there is a growing demand for a reliable cardiac monitoring system to catch the intermittent abnormalities and detect critical cardiac behaviors which lead to sudden death. Use of mobile devices to collect Electrocardiography (ECG), Seismocardiography (SCG) data and efficient analysis of those data can monitor a patient’s cardiac activities for early warning. This paper presents a novel cardiac data acquisition method and combined analysis of Electrocardiography (ECG) and multi channel Seismocardiography (SCG) data. An early warning system is implemented to monitor the cardiac activities of a person and accuracy assessment of the early warning system is conducted for the ECG data only. The assessment shows 88% accuracy and effectiveness of our proposed analysis, which implies the viability and applicability of the proposed early warning system.

## 1. Introduction

In recent past, increasing workload, uneven sleeping patterns and unhealthy life style have degraded human health, particularly cardiac health [1] to a great extent. According to the European heart journal [2], cardiovascular disease is the world-wide leading cause of death across people of all age groups and is responsible for more than 4 million deaths every year in Europe. However, availability of reliable and low-cost healthcare facilities are becoming difficult for common people due to the increasing cost of healthcare services. Although wearable cardiac diagnosis systems are increasingly becoming popular, most people still undergo their routine cardiac checkups using traditional methods such as Echocardiogram (Echo), computerized tomography scan (CT scan), magnetic resonance imaging (MRI) and nuclear myocardial perfusion scan. These methods require hardware and software-based expensive technology and should be handled by professional experts in the hospital. Moreover, such clinical practices are labor intensive, time consuming [3,4,5] and a patient has to visit the hospital for the checkups. Considering the urgency of the problem, there is an increasing demand for inexpensive yet reliable and continuous cardiac health monitoring system.

Recently, Microelectromechanical systems (MEMS) and nanoelectromechanical systems (NEMS) have shown tremendous growth in Mobile Health (mHealth) as a result of which large varieties of low-cost body sensors have been developed to measure various physiological parameters related to human health such as body temperature, heart rate and blood pressure [6,7,8]. The advances of sensor technology have encouraged many to design the sensor-based Body Area Network (BAN) for health monitoring such as activity monitoring [8], drug monitoring [9], diet monitoring [10] and cardiac monitoring [11]. Moreover, most health monitoring systems either employ low-cost wearable devices such as a smart belt, smart band, smart cloth or use smart phone-based sensors to collect the vital body signs, which should be affordable and convenient. Although body sensor-based systems facilitate reliable collection of physiological data, most existing systems are not equipped with data analysis tools to generate the early warnings of cardiac health problems.

In order to monitor the cardiac health related problems [12], there are numbers of clinical practices such as Echocardiogram (Echo), computerized tomography scan (CT), magnetic resonance imaging (MRI), nuclear myocardial perfusion scan and Electrocardiogram (ECG). However, most clinical practices are either highly expensive and require special devices or are not feasible to be designed using body sensors. ECG is realizable via body sensors [13], though it can only measure the cardiac electrical activities, which offer little knowledge on various cardiac mechanical activities such as movement of heart valves, blood circulation into ventricles, suppression-relaxation of ventricle walls, etc. Moreover, stand-alone usage of ECG for cardiac monitoring is neither adequate nor is recommended. According to [14], there are insufficient evidences about the effectiveness of ECG-based diagnosis among people having mild to high risk of Coronary Heart Disease (CHD) problems.

For a person with a healthy heart, cardiac activities take place in a predefined sequence of time at consistent intervals. However, a gradual increase of cardiac related problems such as ischemia, arrhythmias and infarction distorts the consistency of cardiac sequences and changes the cardiac timings. Such abnormalities at mild intensity level are rarely reflected in ECG and therefore other cardiac mechanical vibration recording modalities are required to detect them. Ballistocardiography (BCG) and Seismocardiography (SCG) are known for their ability to record the vibrations generated from various cardiac mechanical activities, which take place between successive heart beats [15]. However, previous studies [16,17,18] have been more inclined towards the SCG than the BCG and there is an increasing consensus on accuracy and applicability of SCG to be considered as an additional measure for clinical purposes. Moreover, recent researches [19,20] demonstrate the viability of collection of SCG using convenient body sensor-based wearable devices. Besides, SCG is a non-invasive method to accomplish the collection of data using inexpensive accelerometer sensors. Due to the lack of reliability of ECG and growing acceptance of low-cost SCG, we are encouraged here to consider SCG as an additional measure to analyze the cardiac ECG and SCG data simultaneously for formulating the early warnings of CHD problems.

The rest of the paper is organized as follows. Section 2 presents the related works along with motivation and goals of our work. Section 3 describes the system model. Section 4 presents various methods to detect the abnormalities in ECG and multi channel SCG data. Section 5 presents the implementation of the proposed early warning system followed by accuracy assessment of the ECG data only. Concluding remarks with future work are given in Section 6.

## 2. Related Works

Nowadays, inexpensive and reliable mobile healthcare systems are increasingly becoming the basic need of a society. In recent years, many efforts have been made to collect [21], communicate [22], store [23] and analyze [24] the healthcare data with a common goal to assist people with their up-to-date healthcare information. Machine learning-based [25], remote cloud-based [26], IoT-based [27] and wearable technology-based [28] approaches are employed in cardiac health monitoring to primarily address the data analysis, storage, visualization and acquisition, respectively. Although existing approaches mainly focus on the specific aspects of the problem, they are not mutually exclusive and require the designing of a complete solution. Among the existing approaches, wearable technology-based cardiac monitoring draws the attention of many researchers as it provides the convenience and reliability of data analysis at low cost. The Human++ [29] is one of the earliest efforts along the direction of Body Area Network (BAN) for diversified health applications, whose primary goal is to monitor and visualize various signals such as electroencephalogram, electrocardiogram and electromyography.

The *LifeGuard* is an e-health monitoring platform designed to monitor health data such as electrocardiogram, heart rate, respiration rate, temperature and blood pressure. The designated system transfers the health data to the base station via bluetooth and proposes buzzer enabled alerts in case of anomalies [30]. The *KNOWME* is a wireless point-to-point body area network enabled sensing platform, which is implemented using off-the-self sensors such as *oximeter, electrocardiograph, accelerometer* and smartphone to monitor as well as analyze various biometric signals round the clock. The motivation behind the *KNOWME* platform is to study the pediatric obesity using in-laboratory and in-field physical activities recorded by the sensors [31]. The *Smart Helmet* [32] proposes a helmet embedded with sensors to continuously monitor the vital signs such as ECG and respiration. It is designed to monitor the health parameters of people engaged in activities such as cycling, motor racing and military. Most existing systems enable 24 h round the clock recording of ECG and heart data. However, such sensor generated data are not readable by humans and therefore may not provide sufficient knowledge to clinicians and researchers in the absence of proper visualization system in place. The *ECG Clock Generator* [33] is an open source data visualization tool for long term monitoring of cardiac activities. The proposed visualization tool formulates an interactive easy-to-interpret plot to distinguish between the healthy and abnormal patterns in ECG for unwieldy large data sets.

The fitting of body sensors directly on body surface is highly inconvenient to the users and therefore *ECG Smart Shirts* [34,35] are designed by weaving electrode sensors along the fabrics to collect unobtrusive ECG cardiac signals. The *PlaIMoS* [36] is an overall architecture comprised of *wearable sensors* for data collection, *IEEE 802.15.4 and IEEE 802.11* data communication network infrastructure, *server* for data analysis and *iOS, Android, Windows 10* applications for data visualization. Most existing cardiac health systems are designed for collecting ECG, heart rate and respiration data due to their ease of retrieval via sensors. However, such systems are less reliable as analyzers of such data do not provide enough in-depth knowledge of complex cardiac activities. To understand the cardiac mechanical activities, Seismocardiography (SCG) is first conceptualized in 1961 and later a novel accelerometer sensor-based technique was proposed by [17] to record the cardiac mechanical vibrations. However, the proposed accelerometer-based method is limited to the laboratory environment. In [19], wearable Seismocardiography is proposed to acquire the cardiac mechanical data for assessing beat-wise cardiac mechanics in ambulant subjects. A smart garment called *MagIC-SCG* is designed for data collection purposes.

In the past, various efforts have been made to ascertain the accuracy and applicability of SCG to monitor the cardiac events. In [16], it is established that cardiac events observed in Echocardiography can also be observed in Seismocardiography as well. Moreover, a set of nine inflection points, i.e., *AS, MC, AO, RE, AC, MO, RF, IM, IC* are also observed in Seismocardiography indicating various unique cardiac mechanical activities [16,17]. This set of inflection points are also called as SCG feature points. In [18] various hemodynamics parameters such as stroke volume, electromechanical systole QS2, pre-ejection period, left ventricle ejection time are estimated. Besides, in [37] a relationship between the myocardial contractility indexes $\frac{dP}{dt_{max}}$ and stroke volume is also established using SCG feature points. However, the combined analysis of ECG and multi channel SCG, which are widely perceived as inexpensive ways to record the cardiac activities have not been studied yet to inform clinicians of the realtime cardiac health conditions well in advance.

### 2.1. Motivation and Goals

Recently, it has been observed that an increasing number of people are dying due to cardiovascular disease. However, there is lack of availability of low cost and reliable cardiac medical services for early warning. The availability of low cost cardiac services are largely based on ECG, which is not considered as a reliable technology and it falls short in terms of monitoring the intermittent cardiac abnormalities. Furthermore, though the body sensor networks-based ECG cardiac monitoring systems accomplish the goal of continuous monitoring of cardiac abnormalities, it still faces the core challenge of reliability and needs further improvements. Besides, reliable cardiac services such as Echocardiography and computerized tomography scan are either expensive, time consuming or not continuous.

To overcome the reliability issues faced by ECG-based systems without increasing the cost and inconvenience, an inexpensive and non-invasive technology needs to be incorporated. Seismocardiography is one of such sensor-based cardiac mechanical motions recording technology gaining popularity and adaptability. However, Seismocardiography is sensitive to vibrations generated by the human motions and respiration and may not help to increase the reliability. To alleviate such problems, Seismocardiography data need to be collected from multiple locations of the heart through multiple channels to reduce the anomalies in data collection. Moreover, this multi channel Seismocardiography and Electrocardiography data need to be analyzed jointly and simultaneously to study the mechanical and electrical behaviors of cardiac activities, which can enhance the reliability of cardiac monitoring. Besides, the result of the SCG and ECG data analysis should be transmitted to the patient in a realtime basis through the early warning system so that a patient can take precautionary measures in advance. The state of art commercial devices such as Shimmer ECG node [38] are used only for collecting the ECG data. In this paper, we focus on collecting the real ECG and SCG data with help of our IRB license and design methods for combined analysis of both ECG and SCG data for detecting the cardiac abnormalities because only ECG data is not reliable. The novelties in our work are the joint collection of ECG and SCG data and combined analysis of ECG and SCG data for abnormality detection with theoretical analysis and evaluation. Since advances in sensor technology have drastically reduced the cost of body sensors and have improved their ability to collect the data with increased accuracy, we are motivated to design the BAN for ECG and SCG cardiac monitoring with early warning system, which can offer a low cost yet reliable solution. The main goals of the our work can be summarized as follows:

1. Design ECG and multi channel SCG data acquisition and communication framework for mobile health monitoring.
2. Develop efficient mechanisms for feature point-based abnormality detection of ECG data.
3. Develop efficient mechanisms for feature point-based abnormality detection of multi channel SCG data.
4. Joint analysis of ECG and multi channel SCG data for cardiac monitoring.
5. Implement the data acquisition and early warning module to collect and visualize the activities of cardiac data.
6. Accuracy assessment of the early warning system for the ECG data is conducted as a case study.

## 3. System Model

**Figure 1:**
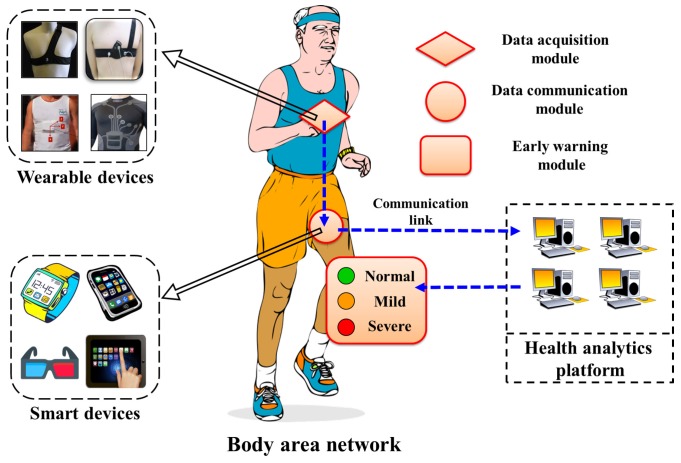
Conceptual architecture of mobile health monitoring system.

The conceptual architecture of Mobile Health monitoring system consists of three different modules namely, (1) Data Acquisition Module; (2) Data Communication Module and (3) Early Warning Module for early warnings of the coronary heart disease as shown in Figure 1. The Data Acquisition Module is meant for collecting the raw ECG and SCG data via various wearable sensors fitted on the human body. The raw data acquired by data collection module are forwarded to the data communication module with help of wireless communication links, which are ultimately forwarded to the cardiac Health Analytic Platform (HAP) located at remote locations. The ECG and multi channel SCG data are processed and analyzed together in the HAP to ascertain the cardiac abnormalities. Based on the frequency and intensity of the detected cardiac abnormalities, HAP generates the set of warning signals and sends them to the early warning module. Upon receiving the signals, the early warning module informs the user about the cardiac health condition such as normal, mild and severe by turning on the corresponding LED lights. The sole purpose of the early warning module is to alert the users in advance to possible future cardiac health problems so that users can consult the cardiac experts/doctors and take preventive measures. The detailed explanation of the functionality of each individual module is described in subsequent subsections.

### 3.1. Data Acquisition Module

The data acquisition module records as well as acquires the location specific ECG and SCG data in a continuous manner. The data acquisition process can be accomplished using Body Area Network (BAN) by fitting various ECG and SCG body sensors to specific parts of the body. Although most ECG and SCG data acquisition methods are laboratory specific, recent advances in sensor technology have made it possible to collect various physiological data round the clock using small and energy efficient wireless body sensors [39,40]. However, it is observed that fitting of such body sensors directly on the surface of the body is highly inconvenient to the users and the real motive of the convenient cardiac motoring system may be not be fully realized. Hence, body sensor-based convenient wearable devices need to be employed.

Recently, wearable devices such as a smart band [41], smart belt [42], smart cloth [19,43] and smart helmet [32] are becoming popular means to collect various physiological data in a continuous manner. There exist plenty of commercial wearable devices, which can successfully retrieve cardiological data. However, it is observed that data collected by most commercially available wearable devices do not fit to the performance analysis due to the lack of reliability and therefore their applications are limited to the introductory diagnosis such as monitoring of heart rate and blood pressure. The core reason behind the limited accuracy of existing wearable devices is their lack of competence in acquiring location-specific data.

**Figure 2:**
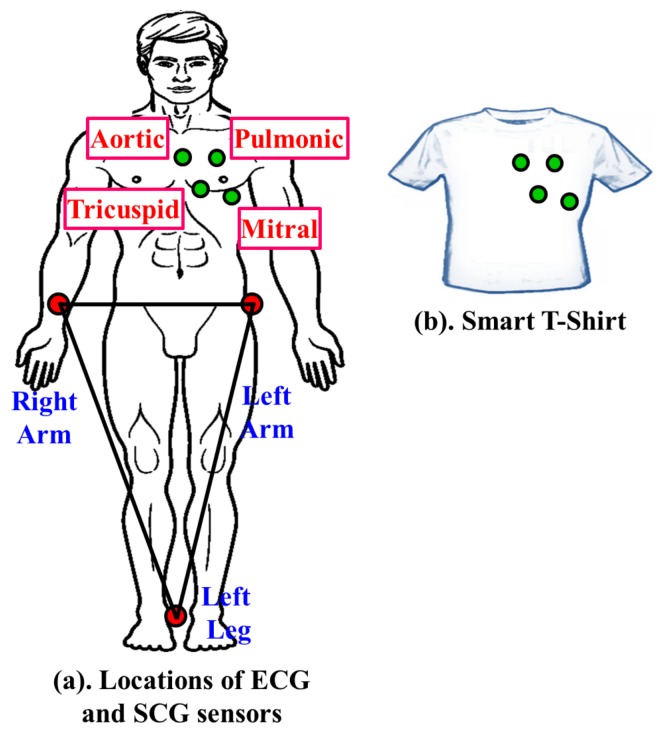
Locations of ECG/SCG sensors for data acquisition.

In the case of cardiological data collection, selection of location of body sensors highly influences the accuracy and quality of the acquired data. Hence, extra attention is paid to choosing the sensor location during the data acquisition phase. Location of various body sensors are chosen-based on the recommendations of the cardiologists. The selected locations of the ECG and SCG sensors in the proposed work are shown in Figure 2a. Three ECG body sensors in the form of electrodes are placed at the left arm, right arm and left leg, respectively. On the other hand, SCG data collection is facilitated by using four accelerometer sensors placed at different valvular auscultation sites such as Aortic, Pulmonic, Tricuspid and Mitral valves, which is also similar to the locations proposed in [12]. In order to ensure the convenience of the users, textile engineers can design a smart suit in the form of a T-shirt by weaving body sensors at specific locations within the fibers as instructed by the cardiologist, which is shown in Figure 2b. It is to be noted that the smart suit can be customized from patient to patient by placing the sensors at accurate locations for collecting the data correctly. Moreover, any sorts of electrical interferences among sensors are avoided by isolating each sensors electrically.

### 3.2. Data Communication Module

**Figure 3:**
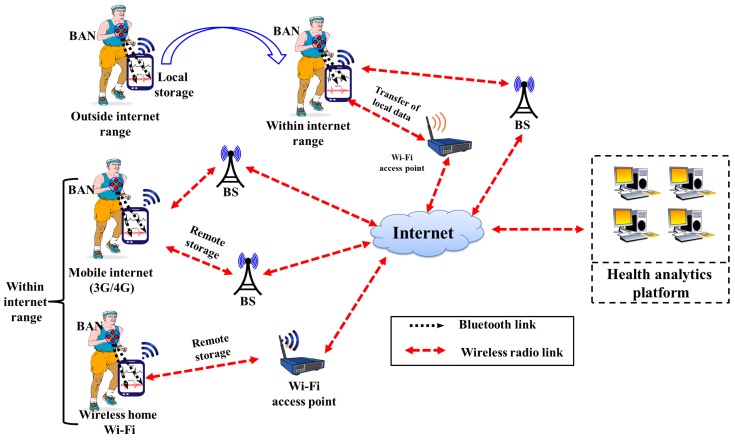
Communication framework between BAN and HAP.

The data communication module acts as a local gateway and is responsible for facilitating the communication between the BAN and HAP. The cardiological data acquired by ECG and SCG body sensors are first transferred to the communication module carried by a mobile user. There are various types of commercially available smart devices such as smart phones, smart glasses, smart watches and tablet-PC, which support the majority of wireless communication protocols such as Bluetooth, 3G/4G and Wi-Fi. Any such smart device that is convenient to carry and adopts the commonly used wireless communication protocols can be considered as a communication module. The communication mechanism between the BAN and HAP is shown in Figure 3. The raw cardiological data are transferred to the HAP, where data are analyzed and corresponding responses in form of the early warning signals are transferred back to the early warning module.

Firstly, ECG and SCG cardiological data are transferred from the data acquisition module to the data communication module with help of the low-power and low-bandwidth communication protocols such as Bluetooth, ZigBee, etc. [44]. From the data communication module, data are either transferred to the HAP for remote storage or are stored locally depending on the location of the user. In order to make the cardiac abnormality detection process reliable and un-interrupted, two broad scenarios are considered based on the user’s location: (1) Within Internet Range; (2) Outside Internet Range. The user is said to be within the internet range if the user is within reach of the WiFi connectivity or cellular network coverage. Subsequently, the cardiological data transmission to HAP takes places via Wi-Fi access point or 3G/4G cellular network Base Station (BS). On the other hand, a user is said to be outside the internet range if the user is not within the reach of a WiFi access point or cellular network coverage area, but within the communication range of Bluetooth.

In order to prevent any loss of data, the collected data has to be stored locally in absence of any internet connectivity, which can be achieved by storing the data in a communication module such as smart phone or tablet-PC carried by the user. The data can be stored temporarily for the duration a user stays away from the internet connectivity. It is to be noted that commonly available smart phones with 16 GB through 128 GB storage capacity can be used for storing the ECG and SCG data temporarily or for longer time duration in case of internet outage. It is assumed that this locally stored data along with the corresponding time stamps are transferred to the HAP as soon as the internet connectivity is established.

### 3.3. Early Warning Module

**Figure 4:**
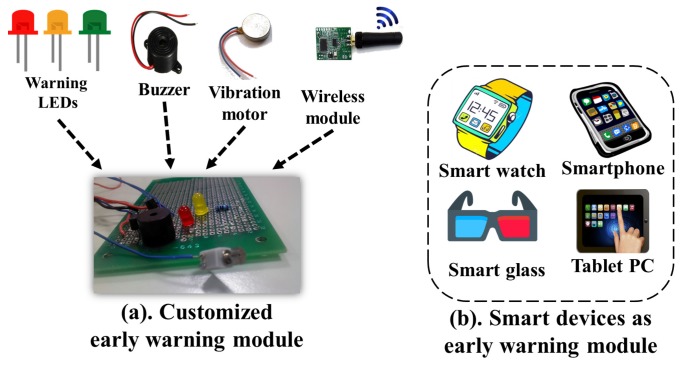
Possible early warning modules.

The Early Warning Module acts as a notifier to the user upon detecting any cardiac abnormality of the ECG and SCG data. The warnings transmitted by the HAP are received by the early warning module and is presented to the user in form of light, sound or vibration. In our proposed design, two early warning modules are shown in Figure 4. We present two approaches to implement the early warning module either by implementing the customized early warning module based on the requirements as shown in Figure 4a or by implementing the customized cardiac monitoring applications for existing smart devices as shown in Figure 4b. The prototype of the customized early warning module is shown in Figure 4a.

The customized module comprises of five basic components namely LEDs to indicate the intensity of the cardiac abnormalities, buzzer to alert the cardiac severity, vibration to generate the mechanical vibrations for indicating the intermittent cardiac abnormalities and wireless module to receive the warning signals from the HAP. The cardiological data analysis is broadly classified into one of the two levels of the cardiac abnormalities namely Mild and Severe. If frequency and intensity of the cardiac abnormalities increase beyond the certain limit, the Yellow LED can glow to indicate the mild cardiac abnormalities. The Red LED glows, if frequent cardiac abnormalities are detected over a user defined time interval to indicate the serious cardiac problems. In addition to the glowing LEDs, the buzzer and vibration are also used to indicate the mild and severe cardiac abnormalities. The hardware-based early warning approach as mentioned above can also be transformed into the software-based implementation as mobile applications in the smart phones. In case of such software-based implementation, buzzers may be replaced with alert ring tones, LEDs may be replaced with interactive charts and descriptive messages.

## 4. Cardiological Data Analysis

In this section, we first introduce various prominent cardiac abnormalities followed by algorithms to detect those abnormalities of the collected ECG and multi channel SCG data.

### 4.1. Abnormality Detection of ECG Data

**Figure 5:**
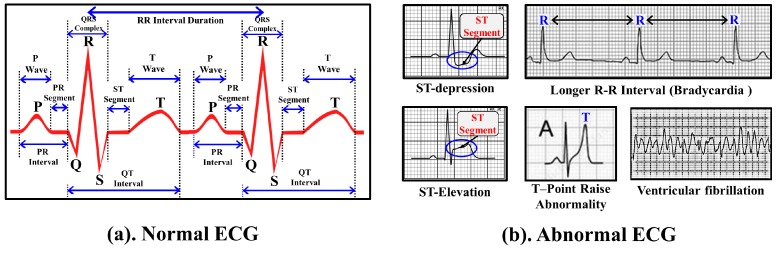
Various cardiac abnormalities in ECG.

**Figure 6:**
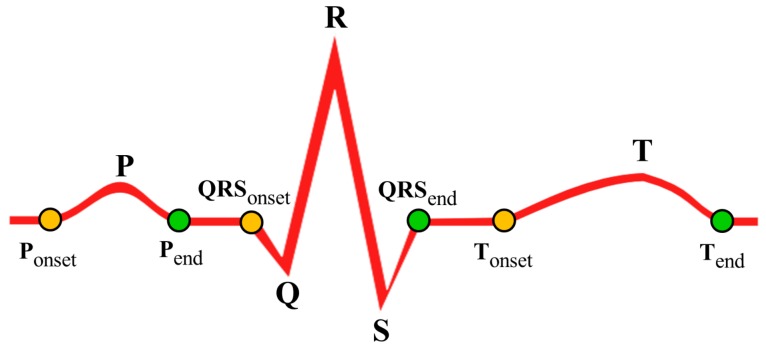
Example of important points, onset points and end points of ECG wave.

ECG is the representation of electrical activities of the heart, which normally take place between two successive heart beats. During normal heart functioning, each ECG cycle represents an orderly progression of depolarization consisting of five important points described as *P*, *Q*, *R*, *S* and *T* as shown in Figure 5a. Successful and accurate extraction of important points helps to detect the cardiac abnormalities in ECG. However, accurate and automatic retrieval of important points is a challenging research issue. A comparative evaluation of ECG delineation method is presented in [45] to retrieve such points. Algorithm 1 describes the overview of the procedure to select the important points *P*, *Q*, *R*, *S* and *T* of the ECG signal. For selecting the peaks, a referenced sliding window SW(X) that contains set of probable data points is derived with respect to the *R* peaks using annotated training ECG cycles, where X={P,Q,S,T}. For a vector of raw ECG data, the set of points with maximum *+ve* amplitude is retrieved after removing the signal artifacts and those points are marked as the candidate *R* points. The rest of the points P,Q,S and *T* are retrieved in each successive *R*–Rduration using the referenced SW(X). Upon retrieval of the important points, set of onset points $P_{onset},QRS_{onset},T_{onset}$ and set of end points $P_{end},QRS_{end},T_{end}$ are retrieved as shown in Figure 6. Using the duration of *P*, QRS and *T* waves observed in normal ECG cycles [46,47], set of onset range $\left\{Range_{onset}\left(P\right),Range_{onset}\left(QRS\right),Range_{onset}\left(T\right)\right\}$ and set of end range $\left\{Range_{end}\left(P\right),Range_{end}\left(QRS\right),Range_{end}\left(T\right)\right\}$ are derived with respect to the important points *P*, *R* and *T*, respectively. Finally, for $P_{onset},T_{onset}$ and $P_{end},T_{end}$, the minimum data points in $Range_{onset}\left(P\right),Range_{onset}\left(T\right)$ and $Range_{end}\left(P\right),Range_{end}\left(T\right)$ are selected, respectively. Similarly, for $QRS_{onset}$ and $QRS_{end}$, the maximum data points in $Range_{onset}\left(QRS\right)$ and $Range_{end}\left(QRS\right)$ are selected, respectively.

Successful extraction of set of important, onset and end points is not enough as it provides little information to conclude the cardiac abnormalities. Hence, various features can be designed based on the position and order of important points such as PRInterval, QTInterval, RRInterval and Segments, i.e., PRSegment, STSegment. Moreover, amplitude of important points and duration of waves can also be considered as important features to detect the cardiac abnormalities. Figure 5a shows various features of ECG data that can be considered to detect the abnormal cardiac activities. One or more ECG features are affected during the abnormal cardiac functioning, which can easily be detected by comparing the value of various features against the standard values observed during the normal ECG. For instance, PRInterval duration may range in between 120 ms to 200 ms under normal cardiac functioning. A PRInterval duration longer than 200 ms may indicates a first degree of heart blocking. On the other hand, a PRInterval duration shorter than 120 ms may indicate the pre-excitation syndrome.

**Algorithm 1: Selection of important points in ECG.**
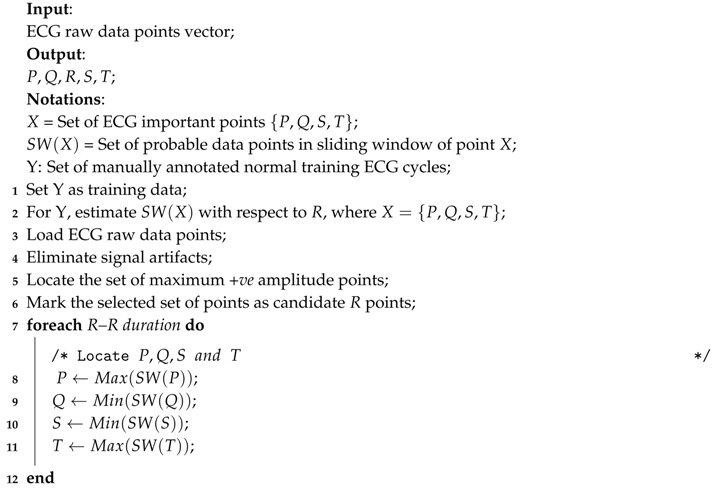


Although existing feature-based methods detect numerous cardiac abnormalities, certain prominent cardiac abnormalities such as ST Depression, ST Elevation, *T* Point raise Abnormality, Longer RR Interval and Ventricular fibrillation as shown in Figure 5b are difficult to detect and therefore designing novel abnormality detection approaches is necessary. In the following subsections, we describe the proposed approaches to detect the cardiac abnormalities for ECG data. It is to be noted that we focus only on prominent cardiac abnormalities and propose-built approaches to detect them. It is to be noted that abnormality detection of cardiac functioning is a complex problem and one approach may not cover and detect all types of cardiac abnormalities. In this paper, we focus our detection only to certain prominent cardiac abnormalities such as ST Depression, ST Elevation, *T* Point raise etc., and subsequently adopt the features such as amplitude, areas and angles to design the respective algorithms.

#### 4.1.1. ST Segment Abnormality Detection

**Figure 7:**
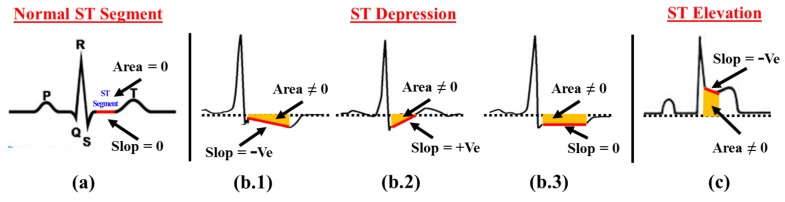
ST Segment abnormality detection method (**a**) Normal *ST* Segment; (**b.1**–**b.3**) *ST* Depression; (**c**) *ST* Elevation.

In a normal ECG cardiac cycle, ST segment can be defined as an isoelectric flat section joining end of the *S* wave, i.e., *J point* with onset point of *T* wave as shown in Figure 7a. For the patients suffering with ventricular ischemia or hypoxia, ST Segment analysis is useful as ventricular ischemia or hypoxia is considered as the leading causes of ST Depression or ST Elevation. Examples of various types of ST depression and ST elevation are shown in Figure 7b.1–b.3, and Figure 7c, respectively. Since, ST segment is a flat isoelectric line, the default slop ($m_{st}$) value of ST segment should be equal to zero. However, the flat ST segment bends towards downward or upward during ST Depression and ST Elevation and accordingly changes the default slop value to −*ve* or *+ve*, respectively.

In order to detect the ST segment abnormality, the proposed method first calculates the slop of the ST segment and ST Depression, i.e., $m_{st}<0$ and ST Elevation, i.e., $m_{st}>0$ is determined based on the slop value $m_{st}$. The value of slop $m_{st}$ for ST Segment can be calculated by finding the angle θ between the baseline and ST segment as defined in Equation (1).

(1) $$\begin{array}{c} m_{st}=\tan\left(\theta \right) \end{array}$$

Although slop $m_{st}$ successfully manages to ascertain the majority of ST segment abnormalities, there are certain cases where the slop value does not change and remains zero in spite of ST depression and ST elevation. One such case for ST depression is shown in Figure 7b.3, where, the slop $m_{st}=0$ in spite of ST depression. Hence, to improve the accuracy of ST segment abnormality detection, we calculate another parameter area ($\sigma_{st}$), which normally forms due to curvature of ST segment with respect to the baseline. In the proposed method, we calculate area ($\sigma_{st}$) by applying the definite integration between *J* point and onset point of *T* wave as defined in Equation (2).

(2) $$\begin{array}{c} \sigma_{st}=\int_{a}^{b}f\left(x\right)dx \end{array}$$

Here, *a* and *b* are points equivalent to *J* and onset point of *T* wave, respectively. f(x) denotes the nature of ST segment curve.

#### 4.1.2. T-Wave Abnormality Detection

**Figure 8:**
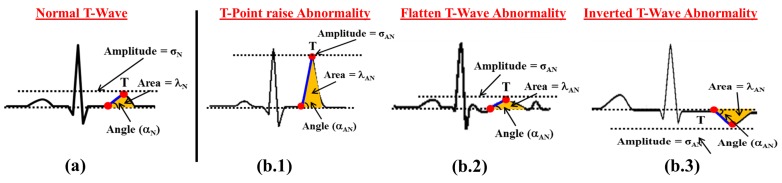
T-Wave abnormality detection method (**a**) Normal T-Wave; (**b.1**–**b.3**) Various T-Wave abnormalities.

Similar to ST segment abnormalities detection, *T*-wave abnormalities detection is also an important and challenging problem. In ECG, the ventricle re-polarization process is reflected in the form of *T*-wave morphology, which provides valuable information to diagnosis coronary ischemia, hyperkalemia and left ventricle hypertrophy disorder. The normal *T*-wave morphology is shown in Figure 8a. There are certain cardiac abnormalities such as *T*-point raise, flattened *T*-wave and inverted *T*-wave that may appear during the ventricle re-polarization as shown in Figure 8b.1–b.3, respectively. Such *T*-wave abnormalities are difficult to uncover using traditional feature-based methods and therefore we propose a novel approach considering the combination of amplitude, area and angle to improve the accuracy of *T*-wave abnormalities detection. For example, when *T*-point raises abnormally, the value of the amplitude, i.e., *peak point*, area and angle of the wave shows abnormally higher values as compared to the values observed during the normal *T*-wave, as shown in Figure 8b.1. In contrast, the *T*-point raises abnormally during flattened *T*-waves; amplitude, area and angle show abnormally smaller or insignificant values. as shown in Figure 8b.2. Finally, the inverted *T*-wave can be detected when amplitude and angle of the wave shows −*ve* value as shown in Figure 8b.3.

#### 4.1.3. RR Interval Abnormality Detection

Another early sign of abnormal heart functioning may be reflected in the form of abnormal heart rates, which is normally reflected in terms of longer RR Interval duration. For an adult, normal resting heart rate may range between 60 BPM to 100 BPM. However, the heart rate falling below 60 BPM can be considered as early signs of Bradycardia, which can be detected by measuring *RR* Interval duration.

The usual RR Interval duration in a healthy heart ranges between 600 ms to 1000 ms. The RR Interval duration longer than 1000 ms can be classified as abnormal. However, it is observed that the heart rate falls below 60 BPM and is perfectly normal to have RR Interval duration longer than 1000 ms while sleeping and sitting. On the other hand, during brisk walking or running, the heart rate increases substantially and observing heart rate lower than 60 BPM with increased RR Interval duration can be considered as serious abnormalities. In order to accurately detect the RR Interval duration related to the abnormalities, we simultaneously observe the user activities when RR Interval duration shows abnormally higher or lower values. The activities are estimated by monitoring the motions of users using accelerometer body sensors.

#### 4.1.4. Other ECG Abnormalities

In addition to the above-mentioned prominent ECG related cardiac abnormalities, we also detect the cardiac abnormalities related to the *P* and QRS wave in terms of amplitude and duration. It is observed that the amplitude $A_{P_{wave}},A_{QRS_{wave}}$ and duration $D_{P_{wave}},D_{QRS_{wave}}$ of *P* and QRS wave range in between certain fixed values for a healthy person as shown in Table 1 [46,47]. The amplitude and duration value of the *P* and QRS wave are calculated from the position and time stamps of important points *P*, *Q*, *R* and *S*. Whenever the value of either amplitude $A_{P_{wave}},A_{QRS_{wave}}$ or duration $D_{P_{wave}},D_{QRS_{wave}}$ varies significantly with respect to the normal values given in Table 1, the abnormality in concerned wave is recorded. It is to be noted that, although combination of amplitude and duration parameters can detect the cardiac abnormalities, it has limitations. In certain cases, normal and abnormal cardiac behaviors may not be distinguished due to inherent variations of normal intervals from one person to another.

### 4.2. Abnormality Detection of SCG Data

Seismocardiography (SCG) is an accelerometer sensor-based method being used to record the cardiac mechanical vibrations. Due to its inexpensiveness, reliability and non-invasiveness, SCG is rapidly gaining popularity and there is a growing consensus among researchers to consider the SCG for clinical practices [16,17,18]. In this paper, SCG is considered as an additional measure along with ECG to monitor and ascertain the cardiac abnormalities with improved reliability.

**Figure 9:**
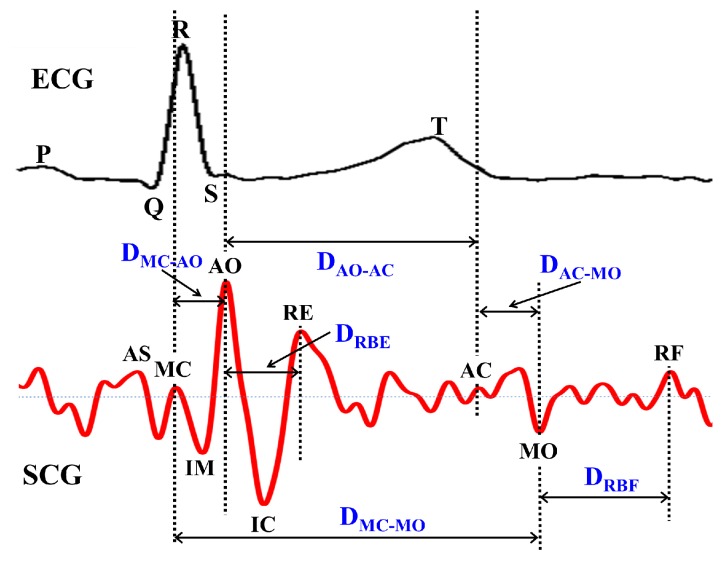
Selection of SCG features for cardiac abnormality detection.

Similar to ECG, SCG exhibits nine prominent important points such as AS, MC, IM, AO, IC, RE, AC, MO and RF as reported earlier in [16]. Algorithm 2 describes the procedure to select the nine SCG important points. For each SCG important point, a sliding window SW(X) is derived with respect to AO using the annotated training SCG cycles, where X={AS,MC,IM,IC,RE,AC,MO,RF}. For the raw data vectors of the SCG, the set of data points with maximum +ve amplitude is retrieved after eliminating the signal artifacts and is marked as AO. The rest of the SCG important points are retrieved for each AO-AO duration using the respective sliding window SW(X). The order of appearance and timing of nine SCG important points with respect to ECG are shown in Figure 9. These nine important points reveal various cardiac mechanical activities such as peak of atrial systole AS, closing of mitral valve MC, isovolumic movement IM, opening of aortic valve AO, isovolumic contraction IC, peak of rapid systolic ejection RE, closing of aortic valve AC, opening of mitral valve MO and peak of rapid diastolic filling RF.

**Algorithm 2:** Selection of important points of SCG.
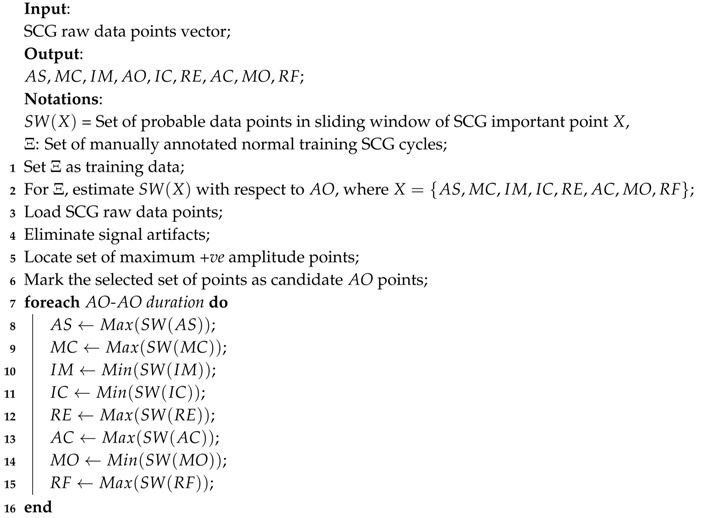


#### 4.2.1. SCG Features Derivation

In order to monitor the cardiac irregularities, six SCG features such as $D_{MC-AO}$, $D_{RBE}$, $D_{AO-AC}$, $D_{MC-MO}$, $D_{RBF}$ and $D_{AC-MO}$ are derived as shown in Figure 9 based on the order and position of nine SCG important points. These features represent the prominent Cardiac Mechanical Activities (CMAs) such as duration between the opening or closing of aortic and mitral valve, systolic blood ejection time, diastolic blood filling time, etc. It is observed that CMAs takes place in specific order with certain time duration for a healthy person. However, coronary heart disease problems such as myocardial ischemia, infarction and arrhythmias impacts the normal operation of CMAs and may significantly change the time duration of various SCG features with respect to those observed during normal functioning. In Table 2, notation for each SCG feature along with corresponding cardiac mechanical activities are listed. The Simple Moving Average (SMA) [48] algorithm is an effective tool that not only smoothens the signal curves and partially filters the signal artifacts but also retains the inherent signal behavior. Before taking the SCG data for analysis, filtering of signal artifacts is carried out using SMA by averaging the data points over five consecutive cardiac cycles.

Unlike ECG, SCG does not have predefined value of duration for various waves. Hence, reference value of duration, i.e., $D_{i}$ for each SCG feature *i*, where 1≤i≤6 is estimated from the predefined δ>0 number of cardiac cycles. The value of δ can be decided based on the recommendation of the cardiologists. In our experiment, we choose δ=20, which gives better performance. From δ number of cardiac cycles, reference moving average duration $\mu (D_{i}^{k})$ and reference moving standard deviation $\sigma (D_{i}^{k})$ are estimated, where 1≤i≤6 and 1≤k≤δ. Calculation of $\mu (D_{i}^{k})$ is given in Equation (3).

(3) $$\begin{array}{c} \mu \left(D_{i}^{k}\right)=\left\{\begin{array}{cc} D_{i}^{k} & if\;\;k=1\\ \mu \left(D_{i}^{k-1}\right)+\frac{(D_{i}^{k}-\mu \left(D_{i}^{k-1}\right)}{k} & if\;\;2\leq k\leq \delta \end{array}\right. \end{array}$$

Here, $\mu (D_{i}^{k})$ represents the estimated average reference duration of *i*th SCG feature in *k*th cardiac cycle. In order to estimate $\sigma (D_{i}^{k})$, continuous variance $S_{i}^{k}$ is estimated using B. P. Welford’s method [49,50], as shown in Equation (4).

(4) $$\begin{array}{c} S_{i}^{k}=\left\{\begin{array}{cc} 0 & if\;k=1\\ S_{i}^{k-1}\;+\left(D_{i}^{k}-\mu \left(D_{i}^{k-1}\right)\right)\;*\left(D_{i}^{k}-\mu \left(D_{i}^{k}\right)\right) & if\;2\leq k\leq \delta \end{array}\right. \end{array}$$

Later, from the continuous variance $S_{i}^{k}$, moving standard deviation $\sigma (D_{i}^{k})$ is estimated as shown in Equation (5).

(5) $$\begin{array}{cc} \sigma \left(D_{i}^{k}\right)={\left(\frac{S_{i}^{k}}{\left(k-1\right)}\right)}^{\frac{1}{2}} & \;\;\;\;if\;\;2\leq k\leq \delta \end{array}$$

**Figure 10:**
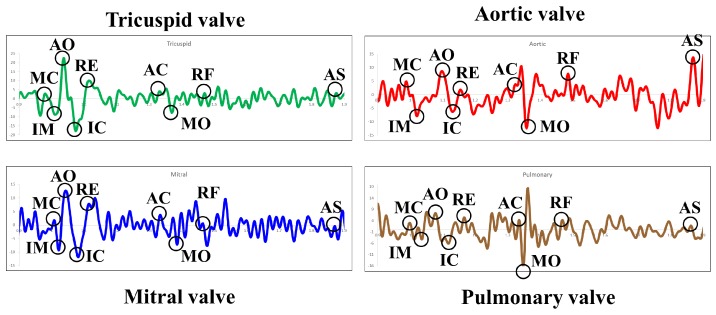
Feature points of multi channel SCG data.

Since, Seismocardiography data obtained through accelerometer sensors is highly sensitive to vibrations generated due to human motion and respiration, the estimated value of $\mu (D_{i}^{k})$ and $\sigma (D_{i}^{k})$ are prone to error. Hence, multi location Seismocardiography values are obtained by placing four accelerometer sensing modules at different valvular sites *Tricuspid valve* TV*, Aortic valve* AV*, Mitral valve* MV *and Pulmonary valve* PV in order to improve the estimation of reference values as shown in Figure 10. For each Seismocardiography signal obtained from different sites, reference moving average duration $\mu {\left(D_{i}^{k}\right)}_{TV}$, $\mu {\left(D_{i}^{k}\right)}_{AV}$, $\mu {\left(D_{i}^{k}\right)}_{MV}$, $\mu {\left(D_{i}^{k}\right)}_{PV}$ is calculated using Equation (3) and reference moving standard deviation $\sigma {\left(D_{i}^{k}\right)}_{TV}$, $\sigma {\left(D_{i}^{k}\right)}_{AV}$, $\sigma {\left(D_{i}^{k}\right)}_{MV}$, $\sigma {\left(D_{i}^{k}\right)}_{PV}$ is calculated using Equations (4) and (5).

Finally, $\hat{\mu (D_{i}^{k})}$ and $\hat{\sigma (D_{i}^{k})}$ are obtained by averaging the value of $\mu {\left(D_{i}^{k}\right)}_{TV}$, $\mu {\left(D_{i}^{k}\right)}_{AV}$, $\mu {\left(D_{i}^{k}\right)}_{MV}$, $\mu {\left(D_{i}^{k}\right)}_{PV}$ and $\sigma {\left(D_{i}^{k}\right)}_{TV}$, $\sigma {\left(D_{i}^{k}\right)}_{AV}$, $\sigma {\left(D_{i}^{k}\right)}_{MV}$, $\sigma {\left(D_{i}^{k}\right)}_{PV}$, respectively as shown in Equations (6) and (7).

(6) $$\begin{array}{c} \hat{\mu (D_{i}^{k})}=\frac{\mu {\left(D_{i}^{k}\right)}_{TV}+\mu {\left(D_{i}^{k}\right)}_{AV}+\mu {\left(D_{i}^{k}\right)}_{MV}+\mu {\left(D_{i}^{k}\right)}_{PV}}{4} \end{array}$$

(7) $$\begin{array}{c} \hat{\sigma (D_{i}^{k})}=\frac{\sigma {\left(D_{i}^{k}\right)}_{TV}+\sigma {\left(D_{i}^{k}\right)}_{AV}+\sigma {\left(D_{i}^{k}\right)}_{MV}+\sigma {\left(D_{i}^{k}\right)}_{PV}}{4} \end{array}$$

The values of $\hat{\mu (D_{i}^{k})}$ and $\hat{\sigma (D_{i}^{k})}$ estimated using training cardiac cycles are used as decision values to find the abnormalities in subsequent evaluation of cardiac cycles. In the evaluation phase, the measured duration of each SCG feature $D_{i}$ is examined with respect to the range $\left(\hat{\mu (D_{i}^{k})}+\hat{\sigma (D_{i}^{k})},\hat{\mu (D_{i}^{k})}-\hat{\sigma (D_{i}^{k})}\right)$ during each subsequent cardiac cycles. If the value of $D_{i}^{j}$ lies outside the range of $\left(\hat{\mu (D_{i}^{k})}+\hat{\sigma (D_{i}^{k})},\hat{\mu (D_{i}^{k})}-\hat{\sigma (D_{i}^{k})}\right)$ in any cardiac cycle *j*, the concerned *i*th SCG feature is considered as potential outlier and the concerned *j*th cardiac cycle is considered as the potential abnormality.

Since the duration of each SCG feature can be assumed as normal distribution with respect to the corresponding average value, Chauvenet’s criterion [51] is employed to calculate the deviation $\varpi D_{i}$ and tolerance $\zeta D_{i}$ for each *i*-th feature of SCG using Equations (8) and (9), respectively. Subsequently, each *i*-th feature of SCG is identified as outlier using $\varpi D_{i}$ and $\zeta D_{i}$.

(8) $$\begin{array}{cc} \varpi D_{i}=\frac{\left|D_{i}^{k}-\mu \left(D_{i}^{k-1}\right)\right|}{\sigma (D_{i}^{k-1})} & \;\;\;\;for\;\;\delta \leq k\leq CCs \end{array}$$

(9) $$\begin{array}{cc} \zeta D_{i}=|NORM.S.INV\left(\frac{1}{4\times k}\right).| & \;\;\;\;for\;\;\delta \leq k\leq CCs \end{array}$$

Here, CCs is the total number of cardiac cycles in evaluation phase and NORM.S.INV represents the inverse of standard normal cumulative distribution. For normal distribution, the statistical rule of thumb suggests that only 5% of data lies outside two standard deviation and should be considered as outliers. Hence, for each *i*-th feature of SCG, the value of $\zeta (D_{i})$ is calculated in such a way that *i*-th feature of SCG whose duration deviates more than two standard deviations with respect to the corresponding average is identified as an outlier.

### 4.3. Combined Analysis of Multi Channel SCG and ECG Data

**Figure 11:**
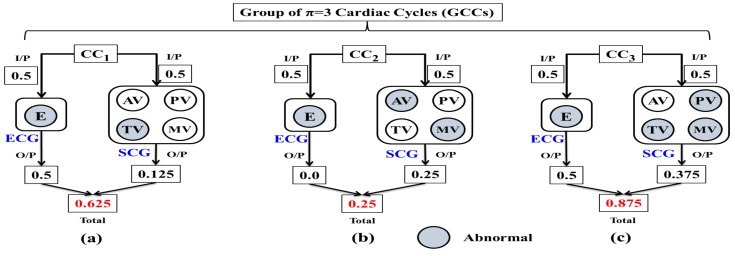
Combined analysis of ECG and multi channel SCG (**a**) Cardiac cycle 1; (**b**) Cardiac cycle 2; (**c**) Cardiac cycle 3.

The combined analysis of ECG and multi channel SCG is needed as both modalities are of Pseudo-accurate. As mentioned earlier, abnormality detection in ECG or SCG cycle does not necessarily conclude the abnormal cardiac cycle. Hence, the probability-based combined analysis is performed to ascertain the chances of any cardiac cycle to be abnormal. Moreover, instead of maintaining the probability of abnormality for individual cardiac cycles, the probability of abnormality for Group of π number of Cardiac Cycles (GCCs) is maintained to find out the intensity of abnormalities over the period of time. Here, the value of π≥1 is user-dependent. Initially, the probability of abnormality of each individual cardiac cycle is calculated, which is later averaged over GCCs. The procedure to find the probability of abnormality for π=3 number of GCCs is shown in Figure 11.

For each modality *ECG and SCG*, the maximum value of the output probability of an abnormality is 0.5. For any cardiac cycle, if both modalities simultaneously result in abnormalities 0.5+0.5=1, the concerned cardiac cycle is marked as abnormal. In case of SCG, the value of output probability of abnormality depends on the outcome of various channels, i.e., *AV, MV, PV, TV*. As shown in Figure 11a, TV detects the abnormality in $CC_{1}$ with output probability $\frac{1}{4}\times 0.5=0.125$ out of four SCG channels. Similarly, the single channel ECG also detects the abnormality with output probability 0.5. Finally, the total probability of abnormality for $CC_{1}$ gives 0.5+0.125=0.625. The said procedure is applied to the subsequent cardiac cycles. As shown in Figure 11b,c, the total probability of abnormality of $CC_{2}$ and $CC_{3}$ is 0.25 and 0.875, respectively. The set of possible values of total probability of abnormality for any cardiac cycle is 0,0.125,0.25,0.375,0.5,0.625,0.75,0.875,1.0. Upon calculating the probability of abnormality for individual cardiac cycles, the probability of abnormality for GCCs is obtained by calculating the average over π number of cardiac cycles. For example, π=3 GCCs and $P_{GCCs}=0.58$.

Finally, $P_{GCCs}$ is compared with the predefined threshold values $\beta_{M}$ and $\beta_{S}$, where $\beta_{M},\beta_{S}\in \left[0,1\right]$ indicates the mild and severe cardiac abnormalities, respectively. For $\beta_{M}\leq P_{GCCs}\leq \beta_{S}$, Yellow LED glows up along with vibration of the motor to indicate the mild abnormalities. Lastly, for $P_{GCCs}>\beta_{S}$, a red LED glows along with the buzzer sound to indicate the severe cardiac abnormalities.

## 5. Implementation

In this section, a case study of the proposed system is presented to assess the accuracy of the ECG abnormality with help of the early warning system. Based on the conceptual system model presented in Section 3, the Data Acquisition Module, Early Warning Module for both ECG and SCG and Accuracy Assessment of Early Warning Module of ECG data are implemented. It is to be noted that the data acquisition module is implemented to collect the multi channel SCG and ECG data simultaneously, and the early warning module is implemented to analyze the ECG and SCG abnormality in Health Analytic Platform (HAP) using our proposed data analysis methods. However, we have considered only the ECG data as our case study in the accuracy assessment module to assess the accuracy of the early warning module. In our implementation, initially we focus on binary classification of the subjects into normal and abnormal category based on the output generated from the HAP, which can be extended to ternary classification with normal, mild and severe categories in future. In order to carry out the experiment, the system model is implemented using various hardware devices and software tools. The detailed description on implementation of all modules is given as follows.

### 5.1. Implementation of Data Acquisition Module

**Figure 12:**
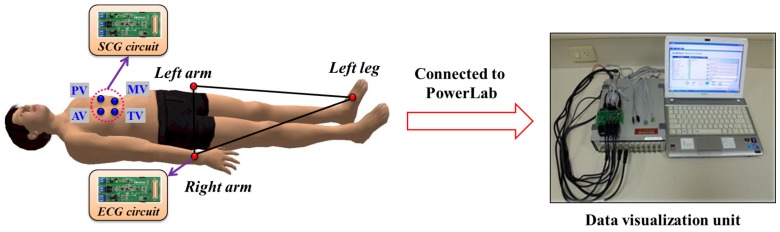
Architectural view of data acquisition module.

The data acquisition module is implemented to collect the single channel ECG and multi channel SCG data simultaneously from different valvular auscultation sites, i.e., *Aortic, Pulmonary, Tricuspid and Mitral*. The architectural view of the data acquisition module is shown in Figure 12. The synchronous ECG and multi channel SCG data collection of 25 subjects are carried out in supine position. To collect the real ECG and SCG data, written consent of these 25 subjects are obtained legally, which is approved by the Institutional Review Board (IRB) of the Chang Gung Memorial Hospital (CGMH), Taoyuan, Taiwan by IRB license number 104-6615B. It is to be noted that license for the data collection procedure was thoroughly reviewed and approved by the IRB committee of the CGMH. In our current investigation, the ECG and SCG signals are collected by putting the subjects in a supine position and without considering any exercising condition such as trade mill test, running or walking condition of a person. However, scope of the experimental trials can be extended to include the mobility and exercising condition of the subjects.

In order to collect the ECG and multi channel SCG data simultaneously, four 3-axis digital accelerometer sensors are employed to get the multi channel SCG waveforms and three electrodes are employed to get the ECG waveforms [12]. Since, accelerometer sensors are highly sensitive to mobility and respiration, the selection of sensors and their subsequent placement on chest surface plays important role in quality of subsequent data acquisition. Hence, we choose the fine quality accelerometer LIS331DLH from STMicro electronics [52] considering its ability to measure the acceleration between 0.5 Hz to 1kHz. Moreover, sensors are placed on the chest surface at specific locations as advised by the cardiologists in such a way that they are isolated enough to avoid the electrical interference and at the same time acquire high quality signal output. On the other side, ECG electrodes are placed at left arm, right arm and left leg to fulfill the requirement of the ECG data acquisition. The placement of accelerometer sensors and electrodes is shown in Figure 12.

In our experiment, the sensing range for all accelerometer sensors are set between the range +2 g to −2 g with 12-bit digital data resolution for better signal quality. Moreover, in order to capture even the micro vibrations generated by various cardiological activities such as blood flow, ventricular movements, opening and closing of the valves, sensitivity of the accelerometers is kept at 1 mg, where *g* indicates the gravitational force. Besides, setting of sensitivity at 1 mg also helps to cancel the external noise up to certain limit. Each accelerometer sensor is embedded into a micro controller SCG circuit system board that consists of ADuC7020 micro controller from Analog Devices Inc (Cambridge, MA, USA) [53]. To log the simultaneous ECG and multi channel SCG data, PowerLab 16/35 from AD Instruments (Dunedin, New Zealand) [54] is used, which is also used to convert the digital data from the micro-controller into analog waveforms. In our experiment, the sampling rate of data collection is kept at 400 Hz. The entire data acquisition process consists of three sub-processes namely the location specific SCG data acquisition, location specific ECG data acquisition and synchronous data logging of ECG and SCG into the system. Although fine quality accelerometer sensors and electrodes are used, it is observed that the acquired data are not completely noise free as some portion of the data are corrupt due to system generated external noise. Hence, the raw signals are first sufficiently amplified and later filtered out before their transfer to the synchronous data logger in order to make them ready for the data analysis. Finally, the acquired clean data from the synchronous data logger are transferred to the host computer for storage, processing, analysis and visualization.

**Figure 13:**
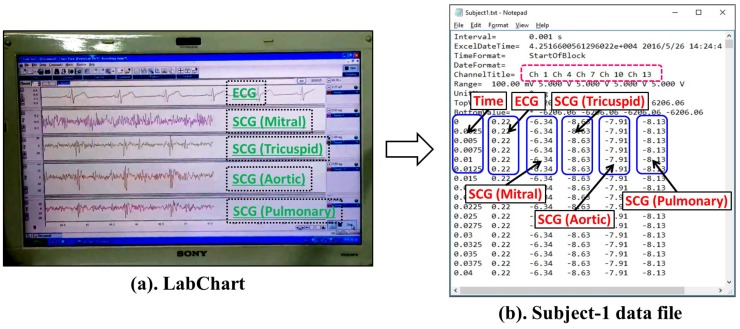
Output of data acquisition module.

**Figure 14:**
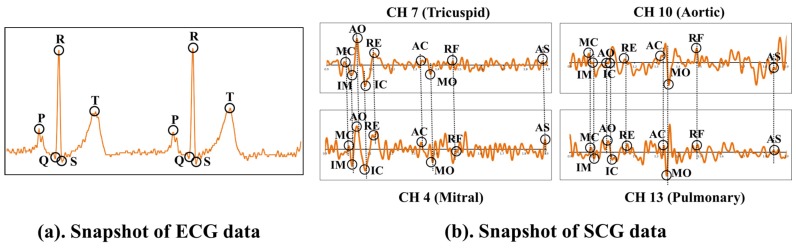
Visualization of ECG and SCG data acquired from PowerLab.

In Figure 13, the output of the data acquisition module is presented. A single channel ECG and multi channel SCG *Mitral, Tricuspid, Aortic and Pulmonary* signals are shown in Figure 13a, which are acquired simultaneously using PowerLab. Figure 13b shows the corresponding storage of data in a file. As shown in Figure 13b, the data file comprises six columns to record the time interval, single channel ECG data and SCG data points collected via four channels. Moreover, Ch 1, Ch 4, Ch 7, Ch 10 and Ch 13 represent the output channel of ECG, SCG (Mitral), SCG (Tricuspid), SCG (Aortic) and SCG (Pulmonary), respectively. Here, Ch represents output channel. Moreover, Figure 14a,b shows the separate ECG and multi channel SCG along with their respective feature points. As shown in Figure 14b, the nine important SCG feature points appear in SCG signals acquired from all four valvular locations, which implies that an efficient data acquisition of SCG is possible from different valvular auscultation sites.

To visualize the analog waveforms of simultaneous ECG and multi channel SCG data from PowerLab, LabChart data analytic platform [55] is used as shown in Figure 13a. Apart from the visualization, LabChart can also be programmed to calculate various parameters such as ECG and SCG feature points.

### 5.2. Implementation of Early Warning Module

Once the data acquisition process is concluded, separate data files consisting of ECG and multi channel SCG data points are generated for each subject. In order to thoroughly validate efficiency of the early warning module, data of 20 subjects are chosen randomly for experimental purpose with equal number of male and female subjects, i.e., *10 subjects per gender* out of the 25 subjects’ data collected from IRB. It is difficult to get the real time abnormal ECG and SCG data of the cardiac patients through our IRB license. On the other hand, inclusion of only healthy subjects, e.g., *false positive* may limit the accuracy of the experimental results and impact the quality. Hence, we have used a programming-based approach to synthesize the data for 30 additional normal and abnormal subjects from the existing set of 20 healthy subjects’ data to balance the number of false positive and false negative. Accordingly, we have synthesized the abnormal ECG data by changing the normal values of amplitude and duration of the important points *P*, *Q*, *R*, *S* and *T* as given in Table 1. For example, the value of normal *P* wave duration and amplitude is 80 ms and 0.1 mm through 0.2 mm, respectively. However, the abnormal value of the amplitude of the *P* wave duration is taken to be more than 120 ms, which indicates the left atrial enlargement abnormality. Similarly, maintaining the *P* wave duration to normal and by changing the value of amplitude of *P* wave to more than 2.5 mm, it indicates the right atrial enlargement abnormality. The snapshot of the demographic of the subjects is shown in Table 3. According to the IRB license, the age ranges between 21 and 28 years for male subjects and the age ranges between 20 and 40 years for female subjects. Moreover, the height, weight and BMI of male subjects ranges between 1.65–1.8 (m), 54–101 (kg) and 18.7–32.6, respectively. Similarly, for female subjects, height, weight and BMI ranges between 1.54–1.69 (m), 45–78 (kg), 18.97–29, respectively. Along with the demographic details, Table 3 also shows the sample data point value of ECG and multi channel SCG in mV. For each subject, data collection is carried out for total 15 min consisting of three sessions of 5 min, each with 5 min of break between the successive sessions. It is to be noted that for each subject, the heart rate and respiratory rate are manually monitored throughout the entire data collection process to ensure the stability and resting position.

**Figure 15:**
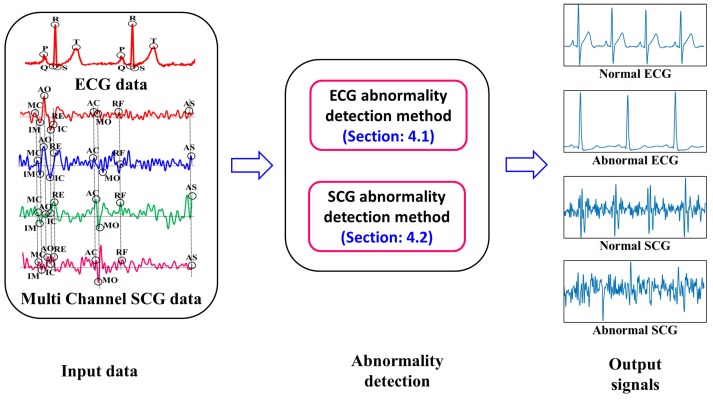
Implementation framework of early warning module.

Taking the output of data acquisition module as input to the Health Analytic Platform (HAP), cardiac health condition of each subject is analyzed subsequently. The implementation framework of Early Warning Module is presented in Figure 15. It is to be noted that simultaneously acquired ECG and multi channel SCG data act as the input to the early warning module. Later, the input data are processed and analyzed using our proposed ECG and SCG abnormality detection methods as described in Section 4.1 and Section 4.2. Ultimately, the output normal or abnormal signals are generated after the analysis of data in HAP as shown in Figure 15.

To visualize the corresponding cardiac health condition of a subject, hardware module is designed using four alarm components such as Yellow LED (Normal), Red LED (Abnormal), Buzzer and Vibration motor as mentioned in Table 4 with their corresponding specification. Based on the abnormality detection of ECG and SCG, probabilistic-based combined analysis as described in Section 4.3 formulate the early warning signals, which are forwarded to the early warning devices to indicate the normal and abnormal cardiac health condition of each subject.

### 5.3. Accuracy Assessment of Early Warning Module

**Figure 16:**
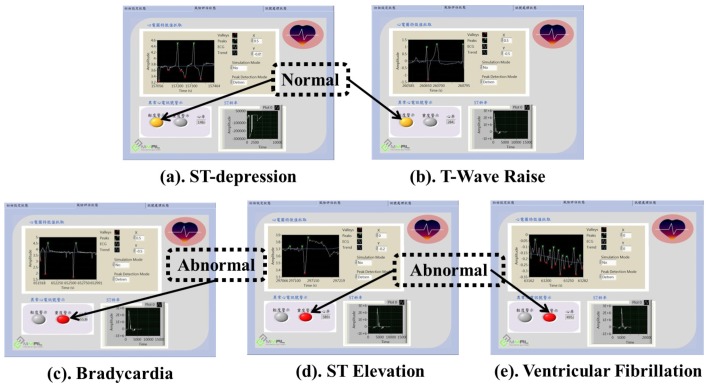
Results of various ECG abnormalities detection.

As mentioned earlier, we have assessed the accuracy of our proposed early warning module taking only the collected ECG data as a case study. Before studying the accuracy of the early warning module, we need to verify the efficiency of the proposed ECG abnormality detection method in detecting various cardiac abnormalities. As shown in Figure 16, various ECG abnormalities such as ST-depression, *T*-Wave raise, Bradycardia, ST Elevation and Ventricular Fibrillation are successfully detected by using the proposed ECG abnormality detection method. It is to be noted that ST depression and *T*-wave raise are of only mild level abnormalities and therefore they are classified as *Normal*, whereas Bradycardia, ST Elevation and Ventricular Fibrillation are of severe level abnormalities and therefore they are classified as *Abnormal* in our case study. Based on the output, i.e., *Normal and Abnormal* generated by the abnormality detection methods, the Yellow LED and Red LED glows up to indicate the Normal and Abnormal cardiac health conditions, respectively. In order to validate the effectiveness of the early warning module, an accuracy assessment of the output generated by the early warning module is also verified by the cardiologists.

**Figure 17:**
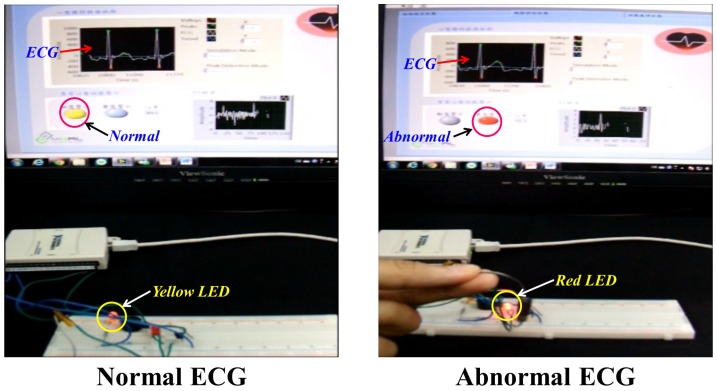
Accuracy assessment result of ECG.

Based on the probabilistic-based combined analysis of the input data as described in Section 4.3, probability of abnormality of group of cardiac cycles $P_{GCCs}$ is calculated taking 10 ECG cardiac cycles as one group. Moreover, calculated value of $P_{GCCs}$ is checked against threshold value $\beta_{S}$ to know the cardiac health condition of the subjects. In this implementation, we chose the single threshold value, i.e., $\beta_{S}=0.6$, which gives maximum accuracy. It is to be noted that probability contribution of ECG and SCG is set to be 1 and 0, respectively as our accuracy assessment is solely based on ECG data. This implies that the Yellow LED of early warning module glows up indicating the normal cardiac condition when $P_{GCCs}\leq 0.6$. On the contrary, when $P_{GCCs}>0.6$, the Red LED of early warning module glows with motor vibration and buzzer to indicate the abnormal cardiac health condition. As shown in Figure 17, the early warning module indicates the glowing Yellow LED for normal ECG. Similarly, glowing of red LED in the early warning module indicates the ECG abnormality.

**Figure 18:**
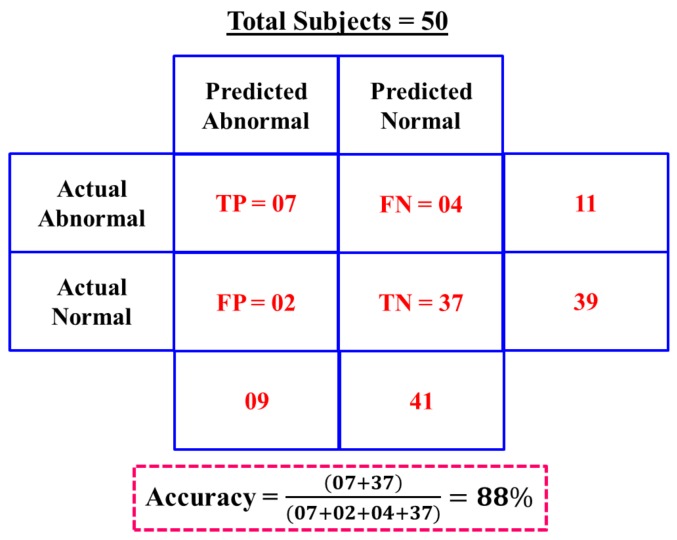
Accuracy result of early warning module.

In the accuracy assessment of our early warning module, 41 subjects are found to be normal out of the 50 subjects with the value of $P_{GCCs}$ between 0.20 through 0.43 and 9 subjects are found to have abnormal cardiac problem with the value of $P_{GCCs}$ between 0.67 through 0.78. The output obtained from the early warning module is considered as the predicted output, i.e., 41 subjects are predicted as normal and 9 subjects are predicted as abnormal. Later, the ECG data of all of the 50 subjects are given to two different cardiologists to classify them into normal and abnormal categories. The ECG data of a subject is considered as normal, when both cardiologists classify the data as a normal subject. Based on the above mentioned exercise, 39 subjects are classified by the cardiologist as normal and 11 are classified as abnormal subjects. To assess the accuracy of the early warning module, the opinions of the cardiologists are considered as actual output and results of the early warning module are considered as the predicted output. Based on the results, confusion matrix as shown in Figure 18 is formulated. Here, TP represents *True positive*, which indicates out of 9 subjects predicted as abnormal, 7 subjects are correctly classified. Similarly, TN represents *True negative*, which indicates that out of 41 subjects predicted to be normal, 39 subjects are correctly classified. On the other hand, FP represents *False positive*, which indicates that 2 are wrongly classified as normal out of 9 subjects predicted as abnormal. FN represents *False negative*, which indicates that 4 subjects are incorrectly classified as abnormal out of 41 subjects predicted as normal. Using the results of confusion matrix, the value of *Accuracy* is calculated using Equation (10) and the high accuracy assessment result 88% shows the efficiency of the early warning module.

### 5.4. Performance Evaluation of Important Points (ECG and SCG)

In this subsection, performance of important points of ECG and SCG as described in Algorithms 1 and 2, respectively is evaluated. Five samples from three normal and two abnormal subjects, each consisting of 20 cardiac cycles are taken into consideration for ECG as well as SCG separately. The description of the sample ECG and SCG data in terms of four parameters is shown in Table 5. The sampling rate of the collected data is 400 Hz. Based on the observed average heart beat rate of the subject and sampling rate, total number of data points in a set of 20 cardiac cycles are derived for each sample. As mentioned earlier, a normal ECG cardiac cycle exhibits five important points, i.e., *P, Q, R, S and T* and an bnormal SCG cardiac cycle exhibits nine important points, i.e., *AS, MC, IM, AO, IC, RE, AC, MO and RF* on a regular basis. In our sample data, the observed number of ECG and SCG important points in a set of 20 cardiac cycles for normal samples are 100 and 180, respectively as given in Table 5. On the other hand, the observed numbers of ECG and SCG important points for abnormal subjects are found to be low compared to normal subjects as given in Table 5. The performance of Algorithms 1 and 2 is evaluated using the sample data shown in Table 5

The performance of both algorithms is evaluated with respect to two performance measures *Accuracy* and *True Positive Rate (TPR)*. The *Accuracy* is an indicator of the overall performance of the algorithms and *True Positive Rate (TPR)* represents the performance ability of the algorithms to correctly select the set of few important points out of the set of large number data points. The *Accuracy* and *TPR* are usually represented in percentage and can be calculated using Equations (10) and (11), respectively.

(10) $$\begin{array}{c} Accuracy=\frac{TP+TN}{TP+TN+FP+FN} \end{array}$$

(11) $$\begin{array}{c} TPR=\frac{TP}{TP+FN} \end{array}$$

Here, TP,TN,FP and FN represents four classification categories, namely *True positive*, *True negative*, *False positive* and *False negative*, respectively, which are normally used to derive various performance measures such as *Accuracy* and *TPR*.

The Algorithms 1 and 2 are executed on the actual data sets as given in Table 5 and the corresponding outcomes are collected as well as analyzed to classify each output data point into one of the four categories, i.e., *TP, TN, FP and FN*. For ECG and SCG, the recorded outcome of Algorithms 1 and 2 in terms of *TP, TN, FP and FN* is presented in Table 6, respectively. From Algorithms 1 and 2, the number of data points classified into TP and TN indicates the number of data points correctly selected as important points and rejected as non-important points (normal), respectively. Higher value of TP and TN gives better performance of the algorithms. On the other hand, FP and FN indicate the number of data points incorrectly selected and are rejected as important and non-important points, respectively. Contrary to the value of TP and TN, larger value of FP and FN indicates the poor performance of the algorithms.

The performance outcome of the algorithms taking four different measures *TP, TN, FP and FN* is merged and the single performance measure *Accuracy* is calculated using Equation (10). Moreover, *TPR* is calculated using Equation (11) to verify the sensitivity of the algorithms in selecting the correct important points. For both ECG and SCG, Table 6 presents the *Accuracy* and *TPR* values for each sample individually along with the average *Accuracy* and *TPR* values over those five samples. The higher outcome of *Accuracy* and *TPR* indicates the efficiency of selecting important points of ECG and SCG algorithms.

### 5.5. Performance Evaluation of SCG Features

In this subsection, we evaluate the quality of the SCG feature set described in Section 4.2. In our evaluation, in total, six SCG features are used for the analysis purpose. To evaluate the quality of SCG features, first we trained each SCG feature using three samples of normal SCG signals, each sample containing number of cardiac cycles. It is to be noted that each cardiac cycle used during training of the features is accurately marked with the position of each of the nine SCG important points as described earlier by expert cardiologists. Further, only normal SCG signals are used to train the SCG features, which effectively estimate the normal time duration of various mechanical activities of the heart. The value of each SCG feature is calculated and is recorded for each of the sixty (3 samples × 20) cardiac cycles and is averaged over to find out the mean and standard deviation of each feature. The output of each SCG feature with mean and standard deviation is shown in Table 7 with *p*-value < 0.05, which indicates the statistical significance of the results.

Once the training phase is concluded, the quality of each SCG feature is evaluated using the test sample set, which comprises five samples of 100 normal and five samples of 100 abnormal cardiac cycles. During the testing phase, all of the six SCG features are calculated for each normal and abnormal cardiac cycle and are compared with the value of trained SCG features to classify the test cardiac cycles into normal or abnormal. The procedure is followed to classify the test cardiac cycles. For each cardiac cycle, the recorded SCG feature value of each feature is compared with the respective trained SCG feature value. If the value of any one of the six SCG features is found outside the range of Mean±SD, the concerned cardiac cycle is marked as abnormal.

**Figure 19:**
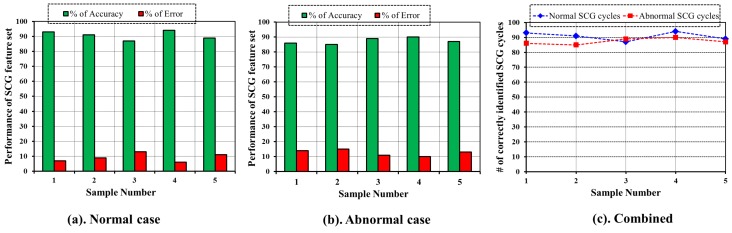
Performance evaluation of SCG feature set (**a**) with respect to normal SCG cycles; (**b**) with respect to abnormal SCG cycles; (**c**) Combination of ECG and SCG cycles.

The outcome of the evaluation procedure is shown in Figure 19. For each sample, performance of SCG feature set is shown with respect to %ofAccuracy and %ofError separately for normal and abnormal as shown in Figure 19a,b, respectively. Moreover, Figure 19c shows the comparison of normal with abnormal case. As observed from Figure 19a–c, the designated set of SCG features has higher accuracy in classifying the cardiac cycle into normal and abnormal, which signifies the quality of the SCG feature set.

## 6. Conclusions and Future Works

Several clinical practices are available to diagnose the reasons behind Coronary Heart Disease (CHD). However, most of the clinical practices are highly expensive and are used to diagnose patients on a non-realtime basis. Further, such practices need special hardware machines and therefore are not suitable to continuously monitor the cardiac conditions. Technical limitation of ECG to record only the cardiac electrical activities makes the diagnosis challenging to gain insights of cardiac functioning. In this paper, a low cost, inexpensive and multi channel Seismocardiography (SCG) data collection and analysis method is explored to strengthen the reliability of existing ECG-based cardiac monitoring systems. Efficient algorithms are designed to locate the abnormalities in ECG and multi channel SCG signals. An intensity and frequency-based early warning module is developed to convey the cardiac health conditions to the users. Separate feature point-based cardiac abnormality detection methods are also incorporated into it. Moreover, probability-based combined analysis of ECG and SCG cardiac cycles is proposed to reduce the error rate of individual cardiac abnormality detection methods and to generate cardiac health indicator-based early warnings. The data acquisition and early warning module for ECG and multi channel SCG and accuracy assessment of early warning module of ECG data are implemented. Experimental results support the objective of viability and applicability of combined analysis of ECG and SCG to design the early warning systems for CHD and the accuracy assessment of both ECG and multi channel SCG will be carried forward in future. However, the proposed system gives a novel direction to generate the early warnings by taking advantage of both ECG and SCG pseudo-accurate methods and achieves significant performance, the psychological factors such as nervousness, excitement and fear factor during the data acquisition and filtering have not been considered, which may affect the accuracy of the analysis. In the future, we may design the data analysis models by incorporating various psychological factors to detect the cardiac abnormalities with improved accuracy.

## Figures and Tables

**Table 1:** Normal values for waves [46,47].

Notation	Meaning
$D_{P_{wave}}$	Normal *P* wave Duration (80 ms)
$A_{P_{wave}}$	Normal *P* wave amplitude (0.1 mm, 0.2 mm)
$D_{QRS_{wave}}$	Normal QRS wave Duration (80 ms, 100 ms)
$A_{QRS_{wave}}$	Normal QRS wave amplitude (≤1 mm)

**Table 2:** Notation and meaning of SCG features.

Notation	Meaning
$D_{MC-AO}$	Time duration from closing of mitral valve to opening of aortic valve.
$D_{AO-AC}$	Time duration between opening and closing of aortic valve.
$D_{MC-MO}$	Time duration between closing and opening of mitral valve.
$D_{AC-MO}$	Time duration from closing of aortic valve to opening of mitral valve.
$D_{RBE}$	Time duration of systolic blood ejection.
$D_{RBF}$	Time duration of diastolic blood filling.

**Table 3:** Demographic snapshot of the subjects.

Subject No.	Gender	Age	Height (m)	Weight (Kg)	BMI	ECG (mV)	SCG Mitral (mV)	SCG Tricuspid (mV)	SCG Aortic (mV)	SCG Pulmonary (mV)
**1**	Male	23	1.71	62	21.2	0.23	−2.01	−1.08	−1.33	−3.27
**2**	Female	27	1.66	57	20.7	0.22	−6.34	−8.63	−7.91	−8.13
**3**	Male	24	1.8	78	24.1	0.62	−0.95	−0.16	−2.68	3.08
**…**	…	…	…	…	…	…	…	…	…	…
**50**	Female	28	1.69	66	23.1	0.33	−2.73	−4.98	2.73	−3.12

**Table 4:** Specification of alarm components.

Component	Specification
**Yellow LED**	Wavelength = 585 nm–595 nm, Emission luminance = 3000–5000 mcd, Voltage = 1.8–2.2 V.
**Red LED**	Wavelength = 620 nm–625 nm, Emission luminance = 1000–1500 mcd, Voltage = 1.9–2.2 V.
**Buzzer**	15 Vp-p 3 mA 80 dB.
**Vibration Motor**	Rate voltage = 3.0 V, Rated current = 60 mA Max, Rated speed = 1400 ± 2500 rpm,
	Stall current = 70 mA Max, Terminal impedance = 40 Ω ± 20%. Stall current = 70 mA Max,
	Terminal impedance = 40 Ω ± 20%.

**Table 5:** Samples of ECG and SCG Data.

	Average Heart Beat Rate	Total # of Data Points	# of ECG Important Points	# of SCG Important Points
$S_{1}\left(N\right)$	82	5854	100	180
$S_{2}\left(N\right)$	63	7619	100	180
$S_{3}\left(N\right)$	71	6761	100	180
$S_{4}\left(AN\right)$	54	8889	91	166
$S_{5}\left(AN\right)$	74	6486	94	171

**Table 6:** Evaluation result of ECG and SCG important points selection algorithms.

	For ECG						For SCG					
	TP	TN	FP	FN	Accuracy	TPR	TP	TN	FP	FN	Accuracy	TPR
$S_{1}\left(N\right)$	92	5709	45	8	0.991	0.92	153	5602	72	27	0.983	0.85
$S_{2}\left(N\right)$	86	7470	49	14	0.992	0.86	159	7356	83	21	0.986	0.88
$S_{3}\left(N\right)$	89	6624	37	11	0.993	0.89	148	6488	93	32	0.982	0.82
$S_{4}\left(AN\right)$	76	8741	67	15	0.991	0.83	134	8642	86	32	0.987	0.80
$S_{5}\left(AN\right)$	81	6334	58	13	0.989	0.86	136	6236	79	35	0.982	0.79
**Average**					**0.991**	**0.87**	**Average**				**0.984**	**0.83**

**Table 7:** Sample value of SCG features.

SCG Features	Mean	Standard Deviation (SD)
$D_{MC-AO}$	0.06	0.012
$D_{AO-AC}$	0.21	0.021
$D_{MC-MO}$	0.30	0.032
$D_{AC-MO}$	0.04	0.010
$D_{RBE}$	0.09	0.011
$D_{RBF}$	0.09	0.019

## Abbreviations

The following abbreviations are used in this manuscript:

TPR	True Positive Rate
TP	True Positive
TN	True Negative
FP	False Positive
FN	False Negative
